# P34 Impact of medical microbiology lead antimicrobial stewardship rounds on antimicrobial consumption in a level 3 hospital

**DOI:** 10.1093/jacamr/dlac004.033

**Published:** 2022-02-16

**Authors:** Mary Lucey, Sabrina O’Regan, Ruth Waldron

**Affiliations:** Department of Microbiology, Galway University Hospital, Galway, Ireland; Department of Pharmacy, Portiuncula University Hospital, Ballinasloe, Ireland; Department of Microbiology, Galway University Hospital, Galway, Ireland; Department of Microbiology Portiuncula University Hospital, Ballinasloe, Ireland

## Abstract

**Background:**

No onsite consultant medical microbiologist (CMM) was available in this centre from September 2018 to November 2019. During this time antimicrobial stewardship (AMS) rounds were carried out by the antimicrobial pharmacist (AMP) with feedback provided directly to clinical teams. Antimicrobial consumption rose from 88.81 DDD/bed-days used (DDD/BDU) in 2017 to 95.57 DDD/BDU in 2019. The aim of this study was to assess the impact of AMS service led by a CMM.

**Methods:**

A structured antimicrobial stewardship programme has been in place since 2013. The re-addition of an onsite CMM in November 2019 facilitated new AMS initiatives, including ward based, targeted antimicrobial rounds, team audits and education sessions led by the CMM. These rounds focused on the choice of antimicrobial agent and duration of therapy.

**Results:**

Overall antimicrobial consumption decreased from 95.57 DDD/BDU in 2019 to 82.16 DDD/BDU (14% reduction) in 2020. The addition of team-based AMS rounds with a CMM and AMP alongside the clinical team enhanced the existing AMS programme and appeared to have the most benefit in reducing antimicrobial consumption. Increases in consumption were noted during weeks where there was poor attendance or cancellation of the AMS rounds.

**Conclusions:**

AMS is most effective with specialty leadership and a multidisciplinary approach. Continuous intervention is required in order to sustain improvements. AMS programmes have been shown to reduce the incidence of healthcare-associated infection and have a positive impact on antimicrobial resistance. AMS should be prioritized by healthcare institutions for increased investment.

